# Serum level of IFNβ distinguishes early from late relapses after biologics withdrawal in rheumatoid arthritis

**DOI:** 10.1038/s41598-022-21160-0

**Published:** 2022-10-03

**Authors:** Eiji Sakashita, Katsuya Nagatani, Hitoshi Endo, Seiji Minota

**Affiliations:** 1 Department of Biochemistry, Jichi Medical University School of Medicine, Tochigi, Japan; 2 Division of Rheumatology and Clinical Immunology, Department of Medicine, Jichi Medical University School of Medicine, 3311-1 Yakushiji, Shimotsuke-shi, Tochigi, 3290498 Japan

**Keywords:** Rheumatoid arthritis, Predictive markers

## Abstract

Since the advent of biological disease modifying anti-rheumatic drugs (bDMARDs) in the treatment of rheumatoid arthritis (RA), most RA patients receiving such drugs have achieved remission at the expense of cost and infection risk. After bDMARDs are withdrawn, a substantial proportion of patients would have relapses even if they were in complete remission. In our previous report, relapse prediction could be made at the time of bDMARD withdrawal by measuring the serum levels of five cytokines. We report herein that, among 73 cytokines examined, serum levels of only interferon β (IFNβ) at the time of bDMARD withdrawal could predict early relapse (within 5 months) in patients who were categorized to relapse by the five cytokines in our previous report, with a cut-off value of 3.38 in log_2_ and AUC of 0.833. High serum levels of IFNβ in the early-relapse group remained high until actual relapse occurred. Therefore, patients who relapse early might be biochemically different from those who relapse late or do not relapse at all. We recommend that patients who are predicted to relapse early continue bDMARDs even if they are in complete remission. This finding contributes to shared decision-making regarding how and when bDMARDs should be discontinued.

## Introduction

While methotrexate remains a mainstay in the treatment of rheumatoid arthritis (RA), newly developed medications have already established their therapeutic position, and even more are emerging to pursue their therapeutic status^1,2^. Such drugs include TNF and IL-6 inhibitors, which are made of antibodies to the corresponding cytokines or their receptors, collectively called biological disease modifying anti-rheumatic drugs (bDMARDs). Another category of drugs is Janus kinase (JAK) inhibitors, which are small molecules that target different intracellular JAKs downstream of a cytokine receptor and therefore inhibit different biologic functions. bDMARDs are historic for their efficacy, and many patients who receive them early in the disease process feel as if their RA is cured. JAK inhibitors might be as effective as bDMARDs in reducing RA disease activity and have the advantage of being administered *per os*. Another advantage, or disadvantage, is their wider target specificity than that of bDMARDs.

The most important and frequently asked question in rheumatology patient care is whether bDMARDs can be discontinued after patients enter remission. Or, are there subsequent relapses after bDMARD withdrawal, and if so, how high is the possibility of relapse^3^? From our previous study and other’s^4,5^, mσRDs after reaching remission, even to the level categorized as Boolean remission, eventually relapse. To discriminate patients who do and do not relapse, our earlier work might provide some clues by calculating the relapse prediction index (RPI) from serum levels of five cytokines^4^.

From Kaplan–Meier curve from earlier reports by other’s and ours, there seems to be two patterns of the relapse mode in RA: early and late relapse^4,5^. Most relapses occur as early as 5 ~ 6 months (early relapse) after bDMARD withdrawal, and the relapse rate after 12 months is very low^4,5^. If distinction between early and late relapse is possible, patients who are categorized as early should strongly be recommended to continue bDMARDs even if they maintain deep remission. To find out potential biomarkers which discriminate early from late relapses, we analysed 73 cytokines in the longitudinal sera from 26 RA patients in our earlier cohort who relapsed from long-term remission after bDMARD withdrawal^4^.

## Materials and methods

### Study design, patients and measurement of cytokines/chemokines

The study design, inclusion criteria for RA patients and the methodology of the measurement of serum levels of cytokines/chemokines were described previously^4^. Briefly, 40 adult patients with RA were prospectively enrolled. The included RA patients had maintained clinical remission in conformity with DAS28-CRP < 2.3 for more than 12 months with bDMARDs (either TNF or IL-6 inhibitors). Then, bDMARDs were withdrawn from the treatment regimen, with other medication remaining unchanged until the end of the study. The patients were evaluated for disease activity, and their serum samples were collected approximately every 4 weeks. They were instructed to visit the clinic any time they felt joint problems. If the disease activity score of a patient became higher than that of a low disease activity score (DAS28-CRP ≥ 2.3), the patient was considered to have relapsed, and the study ended for that patient. If patients stayed in remission, they were followed for 24 months. Measurements of cytokines/chemokines were performed according to the manufacturer’s instructions using the Bio-Plex Pro human chemokine panel (40-plex, Bio–Rad Laboratories, Hercules, CA) and the Bio-Plex Pro human inflammation 1 panel (37-plex, Bio–Rad Laboratories). Both assay kits included heterophilic antibody blocking reagents to inhibit rheumatoid factor interference in the measurements. This study was conducted in compliance with the Helsinki Declaration. The Institutional Review Board of Jichi Medical University approved this study, and patients gave their written informed consent before enrolling in the study. This study was registered in the University Hospital Medical Information Network Clinical Trials Registry (UMIN000044434)*.*

### Statistical analysis

Statistical analyses for the demographics of the patients (Table 1) were performed with EZR version 1.52 (Saitama Medical Center, Jichi Medical University, Saitama, Japan)^6^, which is a graphical user interface for R version 4.02 (The R Foundation for Statistical Computing, Vienna, Austria). The Bio-Plex assay data were normalized by log_2_ transformation for volcano and violin plots, and classical receiver-operating-characteristic (ROC) curve analysis and time-course analysis were performed using GraphPad Prism 9 (www.graphpad.com). Kaplan–Meier survival curves and log-rank tests were used to compare differences between categorical groups. A *p* value less than 0.05 was considered significant. The relapse prediction index (RPI) score was described previously^4^.

**Table 1:** Patient demographics.

Characteristics	Total population (n = 26)		
	Early relapse (up to 6 months) (n = 13)	Late relapse (6 to 24 months) (n = 13)	*P* values
Age, years	59 (44–66)	59 (45–66)	0.959^a^
Female gender, n (%)	11 (84.6)	11 (84.6)	1.000^b^
Disease duration, years	5.0 (4.0–12.0)	7.0 (6.0–11.0)	0.353^a^
Radiographic stage III or IV, n (%)^c^	6 (46.2)	2 (15.4)	0.202^b^
**Number of bDMARDs used before study initiation, n (%)**			0.411^b^
1	7 (53.8)	10 (76.9)	
≥ 2	6 (46.2)	3 (23.1)	
Duration from bDMARD-initiation to remission, weeks	15.0 (12.0–26.0)	14.0 (13.5–16.0)	0.636^a^
Remission duration before study initiation, months	44.0 (33.0–57.0)	56.0 (24.0–62.0)	0.817^a^
Methotrexate, n (%)	8 (61.5)	11 (84.6)	0.378^b^
Dose, mg/week	4.0 (0.0–4.0)	8.0 (6.0–8.0)	0.047^a^
Prednisolone, n (%)	4 (30.8)	0 (0.0)	0.096^b^
Dose, mg/day	0.0 (0.0–1.0)	0.0 (0.0–0.0)	0.037^a^
Seropositive (RF or ACPA) before treatment with bDMARDs, n (%)	13 (100.0)	10 (76.9)	0.220^b^
CRP (mg/dL) before treatment with bDMARDs	0.02 (0.02–0.03)	0.03 (0.02–0.09)	0.075^a^
SAA (μg/mL) before treatment with bDMARDs	0.0 (0.0–0.0)	0.0 (0.0–0.0)	0.823^a^
DAS28-CRP before treatment with bDMARDs	4.25 (3.85–5.17)	3.82 (2.68–4.06)	0.258^a^
DAS28-CRP at study initiation	1.07 (1.04–1.12)	1.13 (1.05–1.21)	0.382^a^
Boolean remission, n (%)	13 (100)	13 (100)	0.609^b^
**TNF inhibitors, n (%)**			0.32
Infliximab	3 (23.1)	6 (46.1)	
Etanercept	6 (46.1)	4 (30.8)	
Adalimumab	0 (0.0)	2 (15.4)	
**IL-6 inhibitor, n (%)**			
Tocilizumab	4 (30.8)	1 (7.7)	

Values are presented as the medians (interquartile range) unless otherwise specified. ^a^Mann–Whitney U test. ^b^Fisher’s exact probability test. ^c^Steinbrocker stage definition. ^d^None of the patients in the late relapse group was taking prednisolone, and 4 in the early relapse group were taking prednisolone at 1 mg in one, 2 mg in two and 3 mg in one at the study initiation. The amount of prednisolone was unchanged throughout the study period. The medians for methotrexate and prednisolone were drawn from all the patients on and off these medications. Statistical analysis was performed using EZR. SAA: serum amyloid A. The upper limit of normal of SAA is 8.0 μg/mL. When the measurement value was < 8.0, the value was assumed to be 0 in this report.

## Results

### Patient Demographics

**Figure 1:**
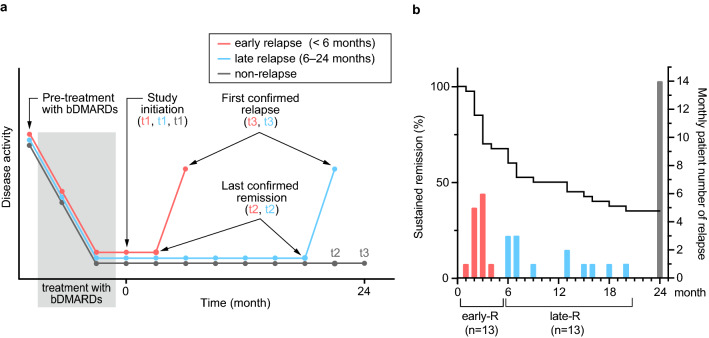
Study design. (**a**) Schema of the timing of clinic visits and serum sampling. The orange–red line indicates a patient group that relapsed earlier than 6 months after bDMARD withdrawal (early relapse). The cyan line indicates a patient group that relapsed within 6–24 months (late relapse). The grey line indicates a patient group with sustained remission for 24 months (non-relapse). t1: time of study initiation (time of bDMARD withdrawal); t2: time of last confirmed remission in relapsed groups or just before study end for a sustained-remission group; t3: time of first confirmed relapse in relapsed groups and study end for a sustained-remission group. The images were created using Adobe Illustrator (ver. 25.4.1, https://www.adobe.com/). (**b**) Kaplan–Meier curve showing the percentage of patients in remission during the 24-month study span and bars showing the number of newly relapsed patients, with orange–red for early relapses and cyan for late relapses. The number of patients in remission decreased quickly during the first 6 months, and the reduction rate became very slow thereafter. Figure 1b was created using GraphPad Prism 9 (www.graphpad.com).

We previously reported a cohort of 40 patients who were treated with bDMARDs and had been in remission for at least a year^4^. bDMARDs used until study initiation were either one of the TNF inhibitors or an IL-6 inhibitor. Remission was originally defined as clinical remission of DAS28-CRP < 2.3; however, all 40 patients were found to have fulfilled the Boolean remission criteria post hoc. bDMARDs were withdrawn from these patients, and they were followed up until relapse was confirmed or up to 2 years in patients who remained in remission (Fig. 1a). Dosing interval and amount of bDMARDs were unchanged until study initiation and prednisolone and conventional DMARDs, if given, were kept used unchanged. Among these 40 patients, 14 maintained remission even 2 years after bDMARD withdrawal, and 26 eventually relapsed at some time point after bDMARD withdrawal. The sustained remission rate in the 40 patients is shown in Fig. 1b along with the actual monthly number of patients who newly relapsed. The slope of the curve was steep during the first 6 months after bDMARD withdrawal and became shallow thereafter. As such, early and late relapses were defined arbitrarily as those that occurred up to 6 months and those that did thereafter, respectively. The 26 relapsed patients were analysed in this report for biomarkers that could potentially discriminate patients who relapse early from those who relapse late. Patient demographics are shown in Table 1. Among 26 patients who eventually relapsed, 13 each were categorized into an early (orange–red) and a late relapse group (cyan) (Fig. 1b). Among the baseline characteristics listed in Table 1, the amount of methotrexate was higher in patients in the late relapse group, and that of prednisolone was higher in those in the early relapse group. There were no differences in other characteristics between the two groups, as shown in Table 1.

### Differences in cytokine profiles between the early and late relapse groups

**Figure 2:**
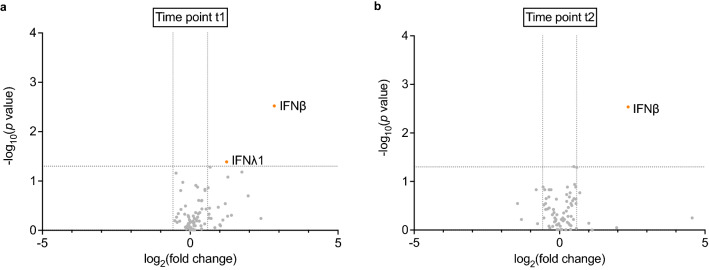
Volcano plot displaying biomarkers with a significant *p value* (y-axis) and fold change (early relapse/late relapse, x-axis) between the early and late relapse groups. (**a**) Volcano plot at time point t1. Biomarkers with ≥ 1.5-fold-change (vertical dotted lines) and *p* value of < 0.05 (horizontal dotted line) are shown by orange–red dots. (**b**) Volcano plot at time point t2. The image was created using GraphPad Prism 9 (www.graphpad.com).

Quantification of 73 cytokines (Table S1) in sera from patients who relapsed early and late was performed with multiplex cytokine/chemokine arrays provided by Bio–Rad Laboratories (Hercules, CA). The assay kits include heterophilic antibody blocking reagents to inhibit the rheumatoid factor interference in the measurements. The patient number of each time point is shown in Table S2. To identify cytokines associated with early relapse after bDMARD withdrawal in RA patients while in remission, serum cytokine levels at time point t1 were compared between patients with early and late relapse (Fig. 2a). Two cytokines, IFNβ and IFNλ1, were found to be higher in patients with early relapse through volcano plot filtering (*p* value < 0.05, fold change > 1.5). At time point t2, only IFNβ was found to be higher in patients with early relapse (Fig. 2b). IFNβ was the only cytokine that was consistently higher in patients with early relapse; therefore, IFNβ was explored further as a possible discriminatory marker between early and late relapses.

### Receiver operating characteristic (ROC) curve analysis

**Figure 3:**
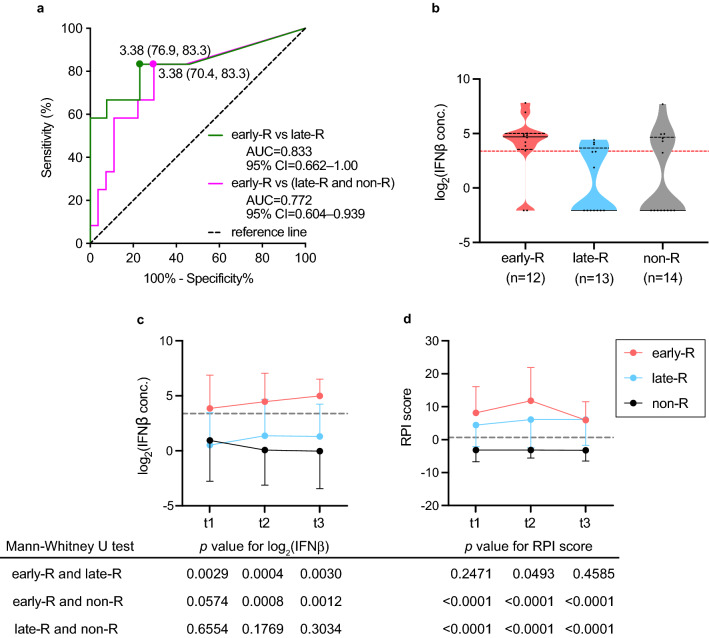
ROC curve and violin plot of IFNβ. (**a**) ROC curves for cut-off derivation against IFNβ. ROC in the green line is based upon early versus late relapse groups, and that in magenta is based upon early relapse versus late plus non-relapse groups. The area under the curve (AUC) for each of the ROC curves is annotated with the 95% confidence interval (CI) by the Wilson/Brown method. The best threshold measured using the “farthest to diagonal line” method with sensitivity and specificity is also shown as a dot on the ROC curve and as an orange–red dotted line in the violin plot. (**b**) Violin plot showing the distribution of the log_2_ transformed level of IFNβ in early (early-R, orange–red), late (late-R, cyan) and non-relapse (non-R, grey) samples. The serum concentration of the analyte was measured in each patient at t1 (Fig. 1a). (**c**) Time-course of changes in log_2_-transformed IFNβ concentrations. Refer to time points t1, t2 and t3 in Fig. 1a. (**d**) Time-course of changes in RPI score. The table indicates *p* values in the Mann–Whitney U test at each time point corresponding to Fig. 3c, d. The images were created using GraphPad Prism 9 (www.graphpad.com).

A ROC analysis was employed to evaluate the discriminatory performance of IFNβ filtered on the volcano plot for early and late relapse. The area under the curve (AUC), specificity, sensitivity, and the optimal cut-off point (log_2_ concentration) values to differentiate early and late relapses at time point t1 were 0.833, 76.9, 83.3, and 3.38, respectively (green line in Fig. 3a). If patients without relapse (n = 14) were combined with those with late relapse, the respective values became 0.772, 70.4, 83.3, and 3.38 (magenta line in Fig. 3a). The distribution of serum IFNβ at time point t1 in the early, late and non-relapse groups is depicted as violin plots, with the cut-off line in dotted red (Fig. 3b).

To evaluate whether IFNβ is an effective biomarker for predicting, in remission, early relapse after bDMARD withdrawal, serum levels of IFNβ were compared in the three groups at three time points, t1, t2 and t3. At each time point, IFNβ was significantly higher in patients with early relapse than in those with late relapse or non-relapse, although the *p* value was 0.0574 by the Mann–Whitney test between the early and non-relapse groups at time point t1 (Fig. 3c). Serum levels of IFNβ did not differ significantly between late and non-relapse groups throughout the time period. The relapse prediction index (RPI) calculated from the 5 cytokines, IL-34, CCL1, IL-1β, IL-2 and IL-19 (cut-off value: 0.63)^4^, nicely discriminated the relapse groups (early and late) from non-relapse at all time points; RPI did not distinguish early from late relapse except at time point t2 (Fig. 3d). These results suggest that the early relapse group is biochemically different from the late relapse group and that IFNβ might be a discriminating cytokine.

### Performance evaluation of the IFNβ-based risk model for early relapse prediction in the entire dataset

**Figure 4:**
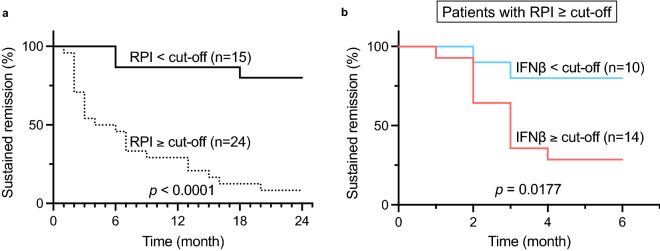
Kaplan–Meier survival curves displaying early relapse estimates in RPI score-positive patients, stratified by IFNβ cut-off value from ROC curves. (**a**) Kaplan–Meier curves for 24-month survival, stratified by the RPI cut-off value of samples at the study initiation point (t1). The solid or dotted graph line represents a group with a low RPI score (n = 15), which predicts sustained remission, or a group with a high RPI score (n = 24), which predicts relapse. (**b**) Kaplan–Meier curves for the first 6 months of survival, stratified by IFNβ cut-off value in a group with a high RPI score. The cyan or orange-red graph line represents groups of low IFNβ score (n = 10) or high IFNβ score (n = 14), respectively. The log-rank test was used to calculate the *p value*. The images were created using GraphPad Prism 9 (www.graphpad.com).

To explore the prognostic value of the IFNβ serum level in RPI-positive patients (predicted to relapse), Kaplan–Meier analysis was performed with the log-rank test using the same dataset. As expected, the RPI-positive patients had a significantly lower rate of sustained remission than the RPI-negative patients (log-rank test, *p* value < 0.0001) (Fig. 4a). Next, the IFNβ-based risk model was applied to determine whether it would have the power to separate the RPI-positive patients into high- or low-risk groups for early relapse with the IFNβ cut-off value of 3.38 in log_2_. Kaplan–Meier analysis for sustained remission during the first 6 months showed a significant difference between the high-risk (≥ cut-off) and low-risk (< cut-off) groups (log-rank test, *p* value = 0.0177) (Fig. 4b). However, Kaplan–Meier curves for the entire 24 months showed that the difference was no longer significant (log-rank test, *p* value = 0.1439) (supplementary Fig. S1), suggesting that the IFNβ-based risk model could only be efficiently applied to early relapse prediction up to 6 months after bDMARD withdrawal in RPI-positive patients.

## Discussion

bDMARDs have shown dramatic treatment efficacy and a much better prognosis in patients with RA. Approximately half of RA patients on bDMARDs with or without methotrexate achieve clinical remission^7–9^. The disadvantage of bDMARDs is their cost and infection risk. Even in Japan, where all residents are covered by health insurance provided by the government, the portion of the cost that must be paid by the patients themselves (10 ~ 30% of the whole costs) is still very high^10^. Many patients forgo bDMARDs due to economic reasons, even if they understand the potent efficacy of bDMARDs in their RA. Another reason for the hesitation is that much is unknown about how long they need to use bDMARDs after they attain remission.

The most common clinical questions patients and physicians want to solve in this context are: 1) how long do patients need to continue bDMARDs? In other words, can patients safely discontinue bDMARDs when they are in remission without uneasiness of future unpredictable relapse? 2) If relapse occurs eventually after bDMARD withdrawal, how early does it happen after withdrawal? and 3) Is it possible to predict an imminent relapse, if any, to resume bDMARDs before patients recognize actual flare-ups? In our previous report to address clinical question 1), we recruited 40 RA patients who had been treated with bDMARDs and entered remission for more than 1 year^4^. bDMARDs were withdrawn, and the patients were followed monthly for 2 years or until relapse occurred. Fourteen patients (35%) remained in remission, whereas 26 patients (65%) relapsed. Five cytokines, IL-34, CCL1, IL-1β, IL-2 and IL-19, and the index (Relapse Prediction Index: RPI) calculated therefrom were found to have predictive power for future relapse, with an AUC of 0.796 at the one-point measurement at the time of bDMARD withdrawal and 0.920 at the two-point measurement including bDMARD withdrawal.

In this report, we sought to answer the second clinical question mentioned above: does relapse, if any, occur early or late after bDMARD withdrawal? As shown in Fig. 1b, the relapse rate was very high; therefore, the line was steep for the first 5 ~ 6 months and became very low thereafter, as judged by the Kaplan–Meier curve and bars of monthly case numbers. Many papers have calculated the relapse-rate using the Kaplan–Meier curve^11,12^. However, observation periods after bDMARD withdrawal were mostly 6 to 12 months, which might be too short to conclude two phases of relapse. A similar line of evidence was shown by a report in which the patients were followed for up to 2 years after bDMARD withdrawal^5^. The commonality between this report and ours is that relapse occurs most often by 6 months of follow-up, and its rate slowed down after that and almost plateaued after one year. The inclusion criteria of the patients in the reports published thus far were also diverse, from low disease activity of relatively short duration to Boolean remission^3,13,14^. In the present study, initial inclusion criterion on disease activity was clinical remission (DAS28-CRP < 2.3). However, after more than 1 year of clinical remission, all the patients including those relapsed early eventually fulfilled Boolean remission at the study initiation, the most stringent remission criterion.

By applying volcano plot analysis at time point t1, serum levels of IFNβ and IFNλ1 were found to have the ability to discriminate patients who relapse early from those who relapse late, although IFNλ1 had only marginal power. At time point t2, only IFNβ showed discriminatory power. To our surprise, serum levels of IFNβ remained high from time point t1 through time point t3, indicating that there might be an inherent difference in the biochemical process between patients who relapse early and those who relapse late. Patients with a high probability of early relapse have better outcomes, in our opinion, if they continue bDMARDs, even if they are in complete remission. Early relapse is often abrupt and severe. Our present report shows that, among patients with highly probable relapses as judged by RPI, the timing of relapse (early vs. late) could be predicted by the serum levels of IFNβ (> 3.38 in log_2_) at the time of bDMARD withdrawal (t1 in Fig. 1a). On the other hand, patients with lower levels of serum IFNβ (< 3.38 in log_2_) might be able to discontinue bDMARDs in the shared decision-making even if RPI predicts future relapses because there would be ample time to follow the clinical course and to prepare for the relapse. Deeper the remission is, less likely the relapse occurs. In our cohort, because the remission depths of all patients were very deep, there was no difference in the prediction ability of IFNβ in the function of DAS28-CRP values.

IFNβ is a cytokine with pleiotropic effects, and its effects in animal models of RA and RA patients are anti-inflammatory in some reports^15–18^. Several clinical trials have included IFNβ as a treatment for RA^19^. The results of the trials were not highly fruitful, and a further trial was not pursued. Conversely, the type I IFN signature is increased in RA^20^ and considered to be proinflammatory^21^. In our cohort, serum levels of IFNβ were higher in the early relapse group than in the late relapse group, indicating that IFNβ could be proinflammatory. The fact that even patients with higher serum levels of IFNβ were all in Boolean remission means that remission criteria might need fine biochemical as well as clinical parameters. Even higher serum levels of IFNβ did not induce clinical inflammatory parameters such CRP or serum amyloid A, indicating that it is working subclinically. Patients who relapse early might be different from those who relapse late in the mechanism of causing and maintaining joint inflammation. According to Fig. 3c, the patients who relapse late might be rather similar in terms of IFNβ to those who do not relapse at all. There was no difference in methotrexate dose between patients with higher and lower serum IFNβ levels. All 4 patients in the early relapse group who were taking prednisolone at 1 ~ 3 mg/day had higher serum levels of IFNβ. Prednisolone dose at this range seems not to show profound effect in the remission level. The time from bDMARDs-initiation to clinical remission was not different (Table 1), either, between the two groups.

IFNα is another major type I IFN, but it was not selected in the present study^22^. Whether there are differences in the pathophysiological action of these two cytokines in RA or a switch-on role of early relapse in RA needs to be elucidated; patients with a high baseline IFNβ/α ratio showed poorer response to TNF inhibitors in RA^23^. Interferon signature was identified in peripheral blood mononuclear cells in early^21,24^ and drug-naïve RA^25^. *SIGLEC1*, one of interferon signature, was expressed in synovial tissue CD68 cells^26^ and Siglec-1 + inflammatory monocytes were increased in the periphery of established RA patients^27^. These results indicate that IFN is proinflammatory and might contribute to RA development. The precise function of IFNβ in inducing early relapse is unknown. However, the similar proinflammatory mechanism of IFNβ might be at work also in relapse with some unknown factors. The intracellular signalling pathways of IFNβ, such as JAK/STAT and beyond, need to be examined to determine its function. To pursue this, cellular components from circulation or joints would be needed, and unfortunately, we did not have these materials for the cohort used in this study. Therefore, the evidence of our current study is circumstantial; higher level of IFNβ is deemed to be a biomarker of early relapse and the cause and effect relationship needs future study.

Mitigation of the obstacles and concerns of RA patients when selecting optimal treatment modalities is undoubtedly important in decision-making. If bDMARDs can be used on and off only when necessary to maintain remission, patients must be encouraged and inspired to use bDMARDs for the lowest cost and risk. We strongly recommend, when deciding bDMARD withdrawal in patients with long-term remission, measuring the serum levels of six cytokines, IL-34, CCL1, IL-1β, IL-2, IL-19 and IFNβ. Next, the relapse prediction index was calculated from the first 5 cytokines to determine whether patients might have high probabilities of relapse^4^. If relapse is highly probable as judged by the index, physicians should discuss with their patients whether to continue or discontinue bDMARDs according to the serum levels of IFNβ. If they are high, early relapse is probable, and it is better to continue bDMARDs. If they are low, relapse could be late in the clinical course after bDMARD withdrawal; ample time would allow meticulous follow-up of patients. Among 14 patients in the non-relapsed group, five had higher levels of serum IFNβ (Fig. 3b). Without sieving by the RPI score, these patients were deemed to relapse early and unnecessary bDMARDs might have continued (Fig. 3b). We think the sieving process by the RPI is necessary in order to increase specificity. However, the measurement of five cytokines to get RPI first is disadvantageous in cumbersomeness and high measurement cost. The confirmation of low IFNβ alone might be enough to expect low probability of early relapses in the practical rheumatology clinic (supplementary Fig. S2). The drawback of our study is the number of patients in our cohort; it is so small that validation is mandatory using sufficiently powered independent cohorts.

Determining predictive biomarkers of imminent relapses while patients are in complete remission is the last and most important clinical question we want to pursue in the future. The present data will help with P4 medicine as proposed by Mucke et al. in rheumatology^28^.

## Supplementary Information

Supplementary Information.

## Data Availability

The data underlying this article will be shared on reasonable request to the corresponding author.
