# Supplementary material for: Serum level of IFNβ distinguishes early from late relapses after biologics withdrawal in rheumatoid arthritis
Source: Sci Rep. 2022 Oct 3;12:16547. doi: 10.1038/s41598-022-21160-0 (PMC9529916; doi:10.1038/s41598-022-21160-0)
Supplement: Supplementary file 1 — Supplementary Information. [file 41598_2022_21160_MOESM1_ESM.pdf]

Table S1. Inflammatory and chemokine biomarkers measured.

Bio-Plex Pro human Inflammation 1 panel		Bio-Plex Pro human chemokine panel	
APRIL/TNFSF13	IL-27 (p28)	6Ckine/CCL21	IL-16
BAFF/TNFSF13B	IL-28A/IFN-λ2	BCA-1/CXCL13	IP-10/CXCL10
sCD30/TNFRSF8	IL-29/IFN-λ1	CTACK/CCL27	I-TAC/CXCL11
sCD163	IL-32	ENA-78/CXCL5	MCP-1/CCL2
Chitinase-3-like 1	IL-34	Eotaxin/CCL11	MCP-2/CCL8
gp130/sIL-6Rβ	IL-35	Eotaxin-2/CCL24	MCP-3/CCL7
IFN-α2	LIGHT/TNFSF14	Eotaxin-3/CCL26	MCP-4/CCL13
IFN-β	MMP-1	Fractalkine/CX3CL1	MDC/CCL22
*IFN-γ	MMP-2	GCP-2/CXCL6	MIF
*IL-2	MMP-3	GM-CSF	MIG/CXCL9
sIL-6Rα	Osteocalcin	Gro-α/CXCL1	MIP-1α/CCL3
*IL-8	Osteopontin	Gro-β/CXCL2	MIP-1δ/CCL15
*IL-10	Pentraxin-3	I-309/CCL1	MIP-3α/CCL20
IL-11	sTNF-R1	*IFN-ϒ	MIP-3β/CCL19
IL-12 (p40)	sTNF-R2	IL-1β	MPIF-1/CCL23
IL-12 (p70)	TSLP	*IL-2	SCYB16/CXCL16
IL-19	TWEAK/TNFSF12	IL-4	SDF-1α+β/CXCL12
IL-20		IL-6	TARC/CCL17
IL-22		*IL-8/CXCL8	TECK/CCL25
IL-26		*IL-10	TNF-α

* IFN-γ, IL-2, IL-8, and IL-10 were measured in both panels.

Table S2. Sampling point and patient number.

Sampling point	Relapse group		Sustained remission group
	early-R	late-R	non-R
t1	12 (1)	13	14
t2	10 (3)	13	14
t3	11 (2)	13	14

Parentheses indicate the number of patient(s) omitted from analyses because of lost samples or poor measurement.

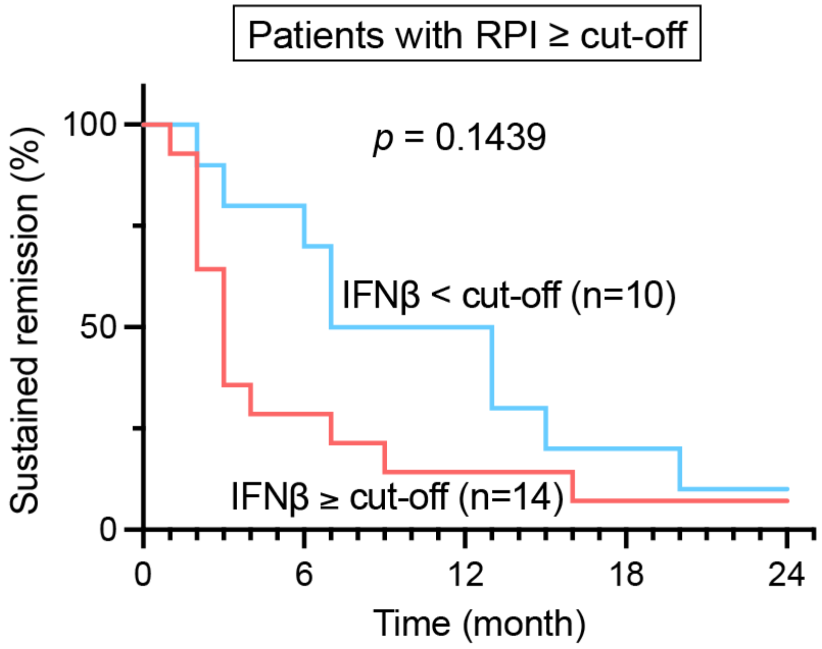

Supplementary Figure S1. Kaplan–Meier survival curves displaying relapse estimates in patients with high RPI scores during the entire 24 months, stratified by IFNβ cut-off value from ROC curves.

The cyan or orange–red graph line represents a group with a low IFNβ score (n =10) or a group with a high IFNβ score (n =14), respectively. The wide difference observed early in the course disappeared as time approached 24 months. The log-rank test was used to calculate the *p value*. The images were created using GraphPad Prism 9 (www.graphpad.com).

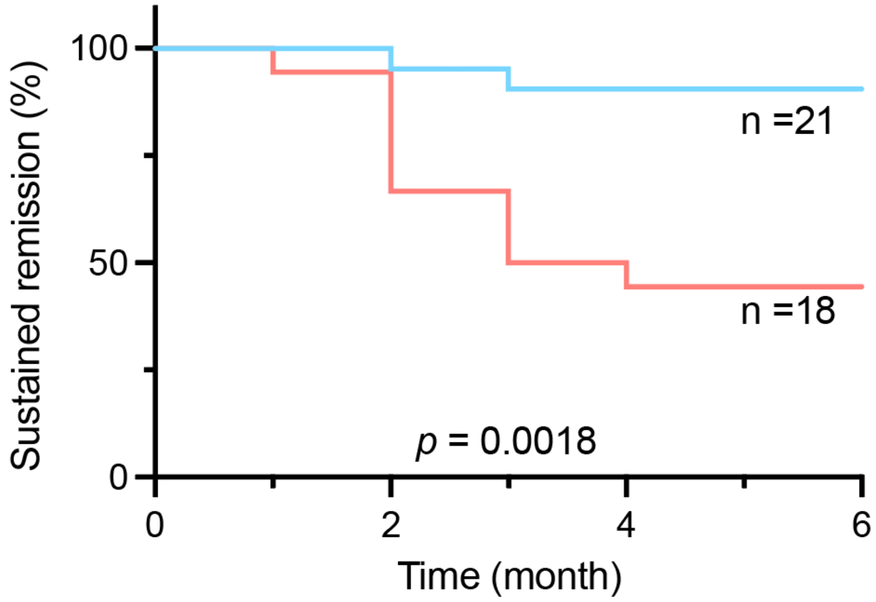

Supplementary Figure S2. Kaplan–Meier curves for the first 6 months of survival, stratified by IFNβ cut-off value in patients with serum measurable by Bio-Plex panels (n=39).

The cyan or orange–red graph line represents groups of low IFNβ score (n =21) or high IFNβ score (n =18), respectively. The log-rank test was used to calculate the *p value*. The images were created using GraphPad Prism 9 (www.graphpad.com).
